# Evolution of the Tn*4371* ICE family: *traR*-mediated coordination of cargo gene upregulation and horizontal transfer

**DOI:** 10.1128/spectrum.00607-24

**Published:** 2024-09-12

**Authors:** Satoshi Matsumoto, Kouhei Kishida, Shouta Nonoyama, Keiichiro Sakai, Masataka Tsuda, Yuji Nagata, Yoshiyuki Ohtsubo

**Affiliations:** 1 Department of Molecular and Chemical Life Sciences, Graduate School of Life Sciences, Tohoku University, Sendai, Japan; Universidad de Buenos Aires, Buenos Aires, Argentina

**Keywords:** mobile genetic elements, LysR transcriptional regulator, integrative and conjugative elements, evolution, PCBs, regulatory evolution

## Abstract

ICE_KKS102_Tn*4677* carries a *bph* operon for the mineralization of polychlorinated biphenyls (PCBs)/biphenyl and belongs to the Tn*4371* ICE (integrative and conjugative element) family. In this study, we investigated the role of the *traR* gene in ICE transfer. The *traR* gene encodes a LysR-type transcriptional regulator, which is conserved in sequence, positioning, and directional orientation among Tn*4371* family ICEs. The *traR* belongs to the *bph* operon, and its overexpression on solid medium resulted in modest upregulation of *traG* (threefold), marked upregulation of *xis* (80-fold), enhanced ICE excision and, most notably, ICE transfer frequency. We propose the evolutional roles of *traR*, which upon insertion to its current position, might have connected the cargo gene activation and ICE transfer. This property of ICE, i.e., undergoing transfer under environmental conditions that lead to cargo gene activation, would instantly confer fitness advantages to bacteria newly acquiring this ICE, thereby resulting in efficient dissemination of the Tn*4371* family ICEs.

**IMPORTANCE:**

Only ICE_KKS102_Tn*4677* is proven to transfer among the widely disseminating Tn*4371* family integrative and conjugative elements (ICEs) from β and γ-proteobacteria. We showed that the *traR* gene in ICE_KKS102_Tn*4677*, which is conserved in the ICE family with fixed location and direction, is co-transcribed with the cargo gene and activates ICE transfer. We propose that capturing of *traR* by an ancestral ICE to the current position established the Tn*4371* family of ICEs. Our findings provide insights into the evolutionary processes that led to the widespread distribution of the Tn*4371* family of ICEs across bacterial species.

## INTRODUCTION

Mobile genetic elements play a crucial role in allowing bacteria to acquire such new traits, including antibiotic resistance, symbiosis, virulence, and compound degradation (1–4). Integrative and conjugative elements (ICEs) are among the various types of mobile genetic elements, which are sometimes referred as plasmids integrated in the bacterial chromosome. ICEs excise themselves from the chromosome, form a plasmid-like circular structure, transfer to a recipient cell by conjugation, and reintegrate into the recipient chromosome (5).

The following are the molecular mechanisms of ICE conjugative transfer, describing using the RP4 plasmid system nomenclature where applicable (6). Integrase (encoded by the *int* gene) with the aid of excisionase (encoded by the *xis* gene) mediates excision by catalyzing site-specific recombination between *attL* and *attR*, which are located at the left and right boundaries of ICE. A set of two sites, *attP* and *attB*, are generated by the recombination: *attP* is on a plasmid-like circular molecule, and *attB* is on a chromosome. The circular form of ICE is transferred to recipient cell by the Dtr (DNA transfer and replication) and MPF (mating-pair formation) systems, which have been extensively characterized in a number of studies on plasmid transfer. In the transfer, *oriT* (origin of transfer) is recognized and nicked by relaxase (encoded by the *traI* gene) to form relaxosome, in which relaxase is covalently bound to the 5' end of DNA. The DNA-relaxase complex is passed to the MPF system by the function of the coupling protein (encoded by *traG*). The MPF system, resembling the type IV secretion system (T4SS), exports the complex of relaxase and single-stranded DNA, which is released as the progression of rolling-circle replication, into a recipient. In the recipient cell, the imported DNA is recircularized, reverts back to double-stranded DNA, and is then integrated into the chromosome by the integrase.

Among diverse ICE families is the Tn*4371* family of ICEs. Tn*4371* is a 54.7 kb transposon carrying genes for PCB/biphenyl degradation (*bph* genes) and was isolated from a mating between *Cupriavidus oxalaticus* A5, which carries the broad host range plasmid RP4, and *Cupriavidus matallidurans* CH34 (7). In a 2003 study, Ariane Toussaint et al. thoroughly sequenced and analyzed the biphenyl catabolic transposon Tn*4371*, revealing its mosaic structure with multiple building blocks, and they proposed the Tn*4371* family as a new family of genomic islands (8). Although Tn*4371* has never been proven to transfer conjugally, the designation of the Tn*4371* ICE family has been used to include a growing number of ICEs (9–12). Ohtsubo et al. compared 112 related elements and showed that βγ-type ICEs (a subgroup of Tn*4371*-related ICEs from β and γ-proteobacterial species) have a distinct conserved structure and are distinguishable from related ICEs from α proteobacteria (αI and αII types). The βγ-type ICEs (Tn*4371* family ICEs) carry four modules, including *int*, *parB*, *traI*, and *mpf* blocks (13).

Tn*4371* family ICEs share the same modular structure. Notably, a *traR* gene, encoding a LysR family transcriptional regulator, is conserved as the first gene of the *mpf* block, which encodes T4SS components (13, 14). This positioning, coupled with its sequence conservation among Tn*4371* family ICEs, suggests its important role in the dissemination of Tn*4371* family ICEs.

**Fig 1:**
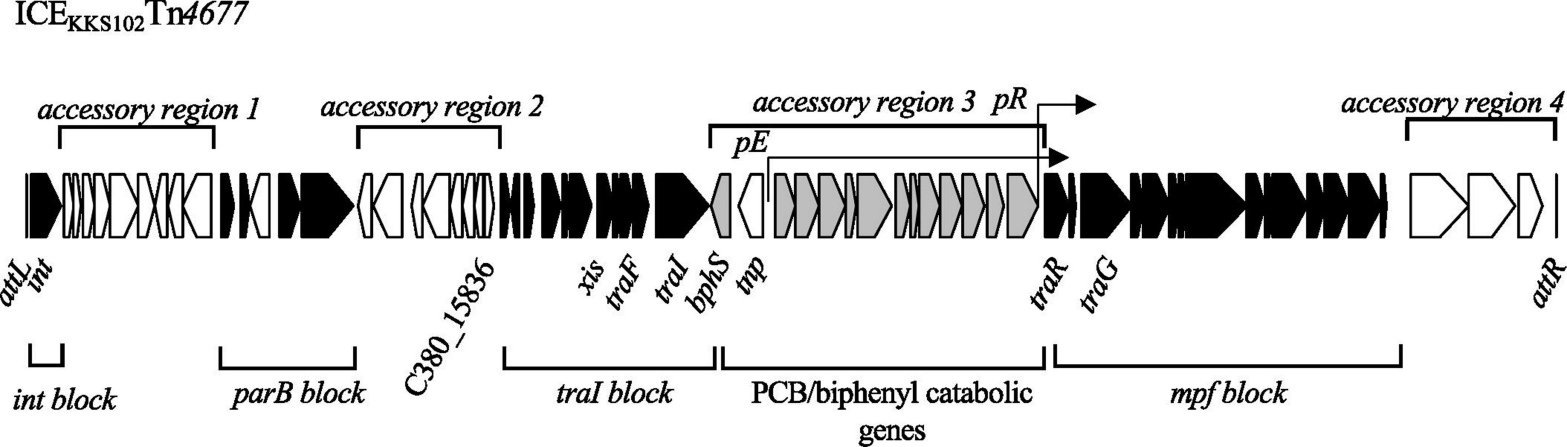
Structure of ICE_KKS102_Tn*4677*. The black arrows represent genes conserved among Tn*4371* family ICEs (13), gray arrows represent PCB/biphenyl catabolic genes, and white arrows represent accessory genes.

The ICE_KKS102_Tn*4677* (Fig. 1) identified from PCB/biphenyl degradative bacterium *Acidovorax* sp. strain KKS102 (15) is the only ICE in the Tn*4371* family for which horizontal transfer has been confirmed (13). ICE_KKS102_Tn*4677* is 61.8 kb in length and located upstream of the Gly-tRNA gene; it is flanked by 9 bp *attL* and *attR* direct repeats (GATTTTAAG). ICE_KKS102_Tn*4677* carries *bph* genes for the degradation of PCB/biphenyl. The expression of the *bph* genes is dependent on the *pE* promoter, whose activity is repressed by BphS through its binding to operator sites overlapping the transcription start site of *pE* (16). The repression by BphS is alleviated by 2-hydroxy-6-oxo-6-phenylhexa-2,4-dienoic acid (HOPDA), a metabolite of the biphenyl degradation pathway (16, 17). The activity of the *pE* promoter is also regulated by favorable organic compounds, and a two-component regulatory system composed of BphP and BphQ is involved in the regulation (18, 19).

The transfer of ICE_KKS102_Tn*4677* to α, β, and γ proteobacterial strains was confirmed, but it occurred at a very low frequency (about 10^−10^ per donor). This low transfer frequency is partly attributed to the low proportion of the ICE being in its excised form (one ICE in the excised form among 10^5^ cells) (13).

In this study, we investigated the role of the *traR* gene in conjugation. We demonstrated that the *traR* gene is transcribed from the *pE* promoter, and the *traR* gene product activates the transfer of ICE_KKS102_Tn*4677* by increasing the proportion of the excised form by activating the transcription of *xis*. The excision alone, however, did not explain the activation of transfer, as *xis* overexpression did not activate the transfer, and the transfer of an *oriT* plasmid required *traR* overexpression. The identification of multiple possible TraR-binding sites suggested a pleiotropic activation function of TraR in conjugation. We discuss the evolutional role of *traR* in the dissemination of Tn*4371* family ICEs based on our findings.

## MATERIALS AND METHODS

### Bacterial strains, plasmids, and growth conditions

The bacterial strains and the plasmids used in this study are listed in Table 1. *Escherichia coli* strains were grown at 37°C in Luria-Bertani (LB) broth (containing 10 g of tryptone, 5 g of yeast extract, and 5 g of NaCl per liter), and the other strains were grown at 30°C in 1/3 LB broth (containing 3.33 g of tryptone, 1.67 g of yeast extract, and 5 g of NaCl per liter). When necessary, antibiotics were used at the following concentrations: 100 µg/mL for ampicillin (Ap), 50 µg/mL for kanamycin (Km), 10 µg/mL for gentamicin (Gm), and 5 µg/mL for tetracycline (Tc).

**TABLE 1:** Strains and plasmids

Strain or plasmid	Relevant characteristics	Source or reference
Strains		
*Acidovorax* sp. KKS102	PCBs/biphenyl degrader carries ICE_KKS102_Tn*4677*.	(20)
SA4	KKS102 derivative carrying the Tc^r^ gene in the 22-nucleotide downstream region of the *bphS* gene.	(13)
SA7	KKS-SA4 derivative, in which the *traR* gene was replaced by the Cm^r^ gene.	(13)
SA10	KKS102 derivative, in which the *bphS* gene was replaced by the Tc^r^ gene.	(13)
SA17	KKS-SA10 derivative, in which the *traR* gene was replaced by the Cm^r^ gene.	This study
H968	KKS102 derivative, in which the *pE* promoter is replaced by the *tac* promoter	(21)
KLZ60	KKS102 derivative carrying *pR*-lacZ fusion in its chromosome. Km^r^	This study
KKS∆S	KKS102 derivative, in which *bphS* gene was inactivated by the Cm^r^ gene insertion	(16)
KLZ60∆S	KKS∆S derivative, carrying *pR-lacZ* fusion in its chromosome. Km^r^	This study
*Pseudomonas putida* KT2440	Type strain; ATCC 47054; Cm^r^	(22)
KT2440Gm	KT2440::TnMod-OGm; Gm^r^	(23)
KT2440ICE	Transconjugant obtained by mating SA4(pNITaraKm_traR) with KT2440	This study
Plasmids		
pKLZ-X	Plasmid for integration of promoter-*lacZ* fusion construct into the genome of KKS102	(16)
pKLZ-W	pKLZ-X derivative with altered MCS	This study
pKLZ60	pKLZ-W derivative for site-specific integration of *pR-lacZ* fusion construct into KKS102	This study
pNIT6012	Tc^r^; shuttle vector able to replicate in *E. coli* and *Acidovorax* sp. KKS102	(24)
pNIT6012dS	Tc^r^; SmaIl-site deletion derivative of pNIT6012	(25)
pNIT6012Km	pNIT6012dS derivative, in which the Tc^r^ gene is replaced by the Km^r^ gene.	(26)
pNITara	pNIT6012dS carrying *araC* gene and *pBAD* promoter from pKD46	(27)
pNITaratraR	pNITara derivative carrying the *traR* gene in its XhoI-EcoRI sites	This study
pNITaraKm	pNIT6012Km derivative, carrying KpnI-NheI fragment carrying the *araC-pBAD* from pNITara	This study
pNITaraKm_traR	pNITaraKm derivative carrying the *traR* gene, excised from pNITaratraR by NheI-HindIII digestion	This study
pNITaraKm_xis.	pNITaraKm derivative carrying *xis*	This study
pNITGm	pNIT6012dS derivative, in which the Tc^r^ gene is replaced by the Gm^r^ gene.	(13)
pNITxisup	pNITGm derivative carrying *xis* upstream region.	This study
pKD46	Ap^r^, oriR101(*repA101*^ts^); λRed recombinase expression plasmid	(28)
pBBR1-MCS5	Low copy number, broad host range plasmid. Gm^r^	(29)
pRebphS	pBBR1-MCS5 derivative carrying the *bphS* gene.	This study
pICEoriT	A pBBR1-MCS5 derivative carrying the *oriT* (*traF-traI* intergenic) region of ICE_KKS102_Tn*4677*	This study
pICEoriT_de1 series	pBBR1-MCS5 derivatives, each carrying a DNA region shown in Fig. 7	This study
pET-22(+b)	A plasmid for protein overexpression in *E. coli*	Novagen
pET22b(+)_His-traR	A pET-22(+b) to express Hisx6-TraR. Ap^r^	This study

### Primers

The primers used in this study are listed in Table S1.

### Construction of plasmids and strains

The construction of a reporter strain KLZ60 is depicted in Fig. S1. A plasmid pKLZ60 was constructed by amplifying a 1 kb *traR*-upstream region carrying the *pR* promoter by using a pair of primers, KKS-pR-F and KKS-pR-R, and ligating the *pR* promoter-containing region to the *lacZ* gene (promoter-less) on a reporter integration plasmid, pKLZ-W. The pKLZ-W was created by digestion of pKLZ-X (16) with EcoRI and BamHI and subsequent ligation of a DNA fragment prepared by annealing a pair of synthetic DNAs, PKLZ-W-a, and PKLZ-W-b (Table S1). The pKLZ60 was used after linearization by HindIII digestion to obtain strains in which the pR promoter-*lacZ* construct was inserted into a specific location (between nucleotide positions 626,209 and 626,718 of the GenBank data with accession number CP003872) in the chromosome of KKS102 by homologous recombination. We obtained KLZ60 and KLZ60∆S by introducing linearized pKLZ60 into KKS102 or *bphS* disruptant (16), respectively. The plasmid pRebphS was created by cloning of a *bphS*-containing fragment into the BamHI and HindII sites of pBBR1-MCS5 (29).

The plasmid pNITaraKm was created by transferring the *araC-pBAD* fragment of pNITara (27) into pNIT6012Km (26). The plasmid pNITaratraR was constructed by inserting a DNA fragment amplified using the primer pair traR-GA-F and traR-GA-R into pNITara (27). The cloned fragment of pNITaratraR containing *traR* was excised by NheI-HindIII digestion and ligated into pNITaraKm to create pNITaraKm_traR. The plasmid pNITaraKm_xis was constructed by cloning of a DNA fragment amplified by the primer pair SM083 and SM084 into pNITaraKm. The pNITxisup was constructed by cloning of a DNA fragment amplified by the primer pair SM103 and SM104 into pNITGm.

A series of pICEoriT plasmids were constructed by cloning PCR-amplified fragments into the EcoRI-HindIII sites of pBBR1-MCS5.

The plasmid pET22b(+)_His-traR for the expression of N-terminal Hisx6-tagged TraR was constructed by amplifying a *traR* gene fragment with a pair of primers, SM141 and SM142, and cloning it into the NdeI-XhoI site of expression plasmid pET-22b(+) (Novagen, Madison, WI).

### Mating experiments

For the mating assay, donor and recipient cells were grown in 5 mL of 1/3 LB medium for 18 h. Subsequently, cells in 1 mL of each culture were harvested by centrifugation at 11,000 rpm for 1 min, washed once with fresh 1/3 LB medium, and mixed and suspended in 50 µL of fresh 1/3 LB medium. This suspension was spotted onto solid 1/3 LB media. To induce expression of the *traR* gene cloned under the *pBAD* promoter, solid 1/3 LB media containing 10 mM arabinose was used. After incubation at 30°C for 24 h, cells were collected using an inoculation loop, suspended in 1/3 LB medium, and then plated or spotted onto solid media containing antibiotic(s) for selection to count mating efficiencies.

When KKS102-derived cells were used for mating experiments, the conjugation efficiency was determined as the number of transconjugants per input donor. In contrast, when KT2440-derived cells were used, the conjugation efficiency was determined as the number of transconjugants per donor counted after mating.

### LacZ activity measurement

LacZ activity was measured as described previously (16).

### Quantification of the circular form of ICE

After strains were grown to the end of the log phase, cells in 1 mL of the culture were harvested by centrifugation and suspended in 50 µL of fresh medium. The suspension was spotted on a solid 1/3 LB medium containing 10 mM of arabinose to induce *traR* expression. After incubation for 12, 18, and 24 h, cells were resuspended in MiliQ water and incubated at 95°C for 10 min. After cells were removed by centrifugation, the supernatants were used as templates for quantitative real-time PCR (qRT-PCR).

qRT-PCR was conducted by using Luna Universal qPCR Master Mix (New England Biolabs, Beverly, MA) and a CFX Connect Real-Time System (Bio-Rad Laboratories, Hercules, CA). The PCR protocol consisted of an initial 2 min at 95°C, followed by 40 cycles of 10 s at 95°C, 10 s at 61.4°C, and 10 s at 68°C. The primer pair SM349 and SM350 was used for the detection of *attB*, SM025-SM026 for *attP*, and SM026-SM027 for both the integrated and excised forms. For each sample, the three sets of primers were used. The results obtained with SM026-SM027 served as an internal standard to normalize the differences in the amount of DNA used for the qRT-PCR.

### Quantification of the expression of genes in ICE_KKS102_Tn*4677*

RNA in each sample was extracted by ISOGEN (NIPPON GENE, Tokyo, Japan) according to the provided protocol. After extraction, each RNA sample was treated with DNase I (TAKARA, Shiga, Japan) at 37°C for 2 h to remove the residual DNA. Reverse transcription was conducted by using ReverTra Ace︎ qPCR RT Master Mix (Toyobo, Osaka, Japan) and random 9-mer primers.

The qRT-PCR was conducted as described above for the detection of *attP* and *attB*. The primer sets SM015-SM016, SM019-SM020, SM041-SM042, SM105-SM106, and SM142-SM143 were used to quantify *traR*, *rrn*, *traG*, *xis*, and *traI*, respectively. The expression levels of each gene were normalized to the expression levels of the rRNA gene, *rrn*.

### Purification of TraR

TALON Metal Affinity Resin (TAKARA, Shiga, Japan) was used to purify N-terminal Hisx6-tagged TraR protein according to the provided protocol.

### Gel shift assay

The FAM-labeled *xis*-upstream-region DNA was prepared using the primer pair PNIT5041-FAM and PNIT5548-FAM with pNITxisup as a template. The FAM-labeled *oriT*-region DNA was prepared using the primer pair M4out-FAM and RVout-FAM with pICEoriT as a template. The FAM-labeled *traG*-upstream-region DNA was prepared using the primer pair SM484 and SM485-FAM with genomic DNA of KKS102 as a template. For the preparation of DNA containing the TraR-binding motif, the following pairs of oligonucleotides were mixed and annealed: X001-X002 (BSxis), X003-X004 (BStraG), X005-X006 (BStraI), and X007-X008 (BStraR).

TraR with an N-terminal Hisx6 tag was expressed in BL21(DE3) cells containing the expression plasmid pET22b(+)_His-traR and purified using Ni-NTA agarose (Qiagen, Hilden, Germany). The purified protein was mixed with purified DNA fragments (90 nM) in a buffer containing 40 mM NaCl, 80 mM Tris-HCl pH 8.0, and 5 mM MgCl_2_. The final concentrations of the protein were 2, 5, and 10 µM. Samples were electrophoresed on a 15% polyacrylamide gel at 200V for 60 min using Tris-glycine buffer. Detection was performed using the FLAS-T1500 system (Fujifilm, Tokyo, Japan).

### Colony PCR

To confirm that the colonies grown on plates containing Tc and Gm carried ICE_KKS102_Tn*4677*, colony PCR was conducted using the KOD-One PCR master mix (Toyobo). Two sets of primers, SM021-SM022 and SM023-SM024, were used to amplify DNA fragments of 3,058 bp and 1,087 bp in length, respectively.

### RNA-seq

Cells were cultured to the end of the log phase, and arabinose was added to induce *traR*. After 1 h, cells were harvested, and RNAs were prepared using the RNeasy Mini Kit (QIAGEN). The prepared RNA samples were then sent to GeneWith (Yuseong-gu, Daejeon, Korea) for TruSeq sequencing.

Obtained next-generation sequence (NGS) reads were mapped using our in-house mapping tool, NGSPairMapper, available on GitHub (https://github.com/ohtsuboy/NGSPairMapper). NGSPairMapper uses two initial 21-mers from a pair of reads and searches for mapping locations based on 100% identity. It maps read pairs with an end-to-end distance of 20–2,000 base pairs, selecting the shortest distance when multiple mapping locations are found. The genomic range spanned by the pair was counted as a transcript. NGS read data were submitted to the Short Read Archive of the National Center for Biotechnology Information under the accession numbers SAMN42563764, SAMN42563765, SAMN42563766, and SAMN42563767.

## RESULTS

### *traR* belongs to the *bph* operon, and its transcription is regulated by *bphS*

**Fig 2:**
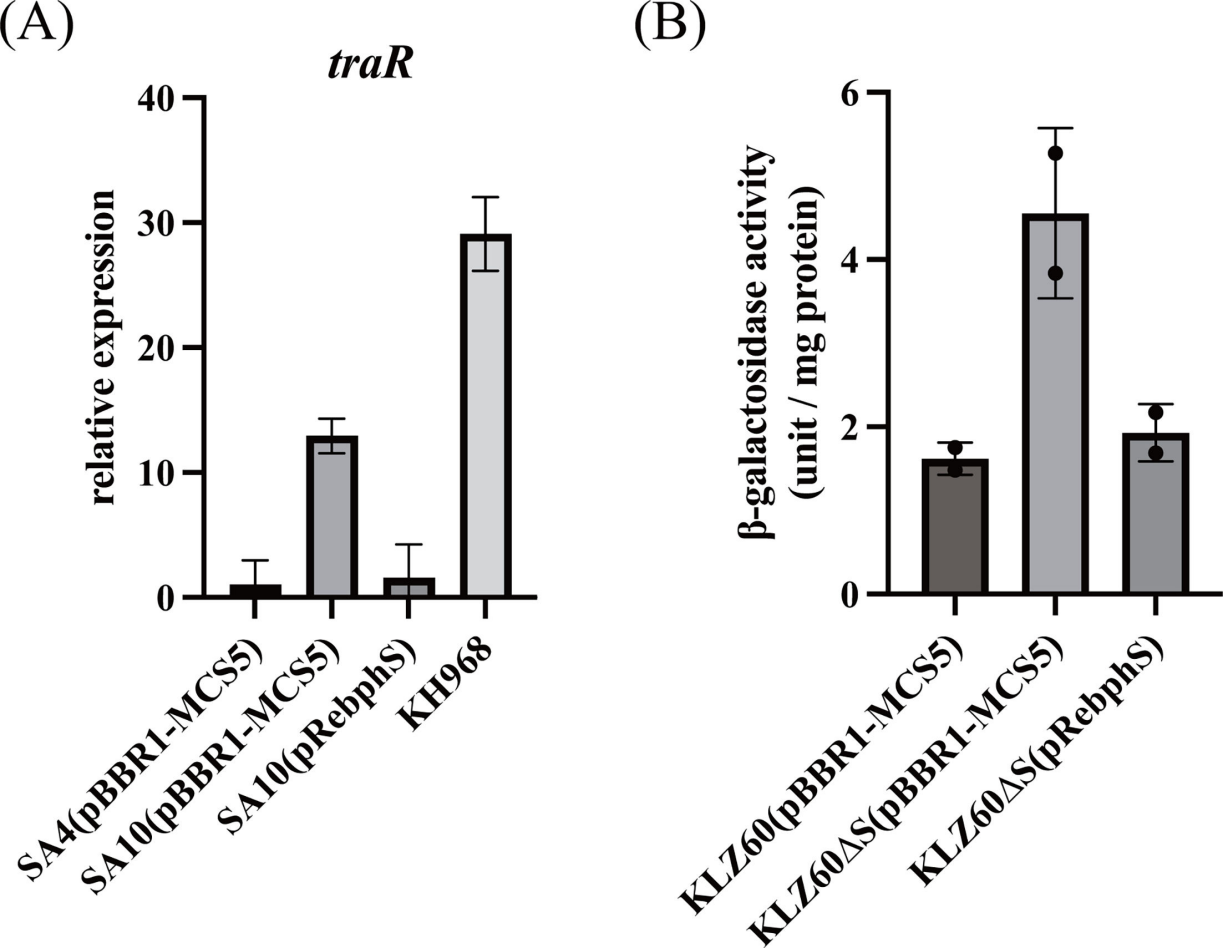
*traR* belongs to the *bph* operon. (**A**) qRT-PCR assay showing that *traR* belongs to the *bph* operon. Each strain was grown to an OD600 value of 0.8 in 1/3 LB medium, and then total RNA was isolated for qRT-PCR analysis. The values are relative to those of the strain SA4(pBBR1-MCS5), and the *rrn* level was used as an internal standard. (**B**) LacZ reporter assay showing that the *pR* promoter level is increased in ∆*bphS*. LacZ activity is expressed as unit/mg protein (16). Each strain was grown as in panel A.

We predicted that *traR* is part of the *bph* operon because it is located 294 bp downstream of *bph* genes in the same orientation, and no transcriptional terminator sequence was predicted. To test this, we conducted qRT-PCR to measure the *traR* transcription levels in strains SA4 and SA10 (Fig. 2, panel A). The strain SA4 is identical to the wild-type strain KKS102, except that it carries a Tc resistance gene inserted downstream of the *bphS* gene. The strain SA10 is a KKS102 mutant, in which the *bphS* gene is replaced by the Tc resistance gene. The qRT-PCR analysis revealed a 13-fold increase of *traR* transcripts in SA10 (pBBR1-MCS5) compared with SA4 (pBBR1-MCS5), and this increase was suppressed by complementing SA10 with pRebphS, a pBBR1-MCS5-derived plasmid carrying *bphS*. We also tested a strain KH968, in which the *pE* promoter for the *bph* operon is replaced by a constitutive *ptac* promoter (21, 30). Similarly, *traR* transcription was elevated 30-fold in KH968. These observations showed that *traR* is part of the *bph* operon and transcribed from the *pE* promoter.

### *The pR* promoter for *traR* transcription is upregulated in ∆*bphS*

To test the possibility that *traR* has its own promoter and, if so, whether that promoter is regulated by *bphS*, a 1 kb upstream region of *traR* was ligated in front of the *lacZ* gene (promoter-less) and integrated into the genome of KKS102 (resulting in a strain KLZ60; Fig. S1) to measure the promoter activity of this region (Fig. 2, panel B). We found that the LacZ activity was increased when the *bphS* gene in KLZ60 was deleted (strain KLZ60∆S), and this increase was suppressed by supplying the *bphS* gene in *trans*. These results indicated that a promoter is present in front of *traR* (designated as the *pR* promoter), and *pR* is negatively regulated by BphS. However, we found no sequence similar to known BphS-binding sites (16) in the 1 kb region.

Because a number of LysR-type transcriptional regulators are known to control its own transcription (31), we speculated that the high *pR* activity in KLZ60∆S was due to *traR* upregulation. To test this, KLZ60 was transformed with the *traR* expression plasmid pNITaraKm_traR, which carries the *traR* gene, under the arabinose-inducible pBAD promoter and tested for LacZ activity. The effect of *traR* was tested by using cells grown on a solid medium. However, we were unable to obtain any results showing that *pR* is upregulated by TraR.

### *traR* overexpression activates the transfer of ICE_KKS102_Tn*4677*

**Fig 3:**
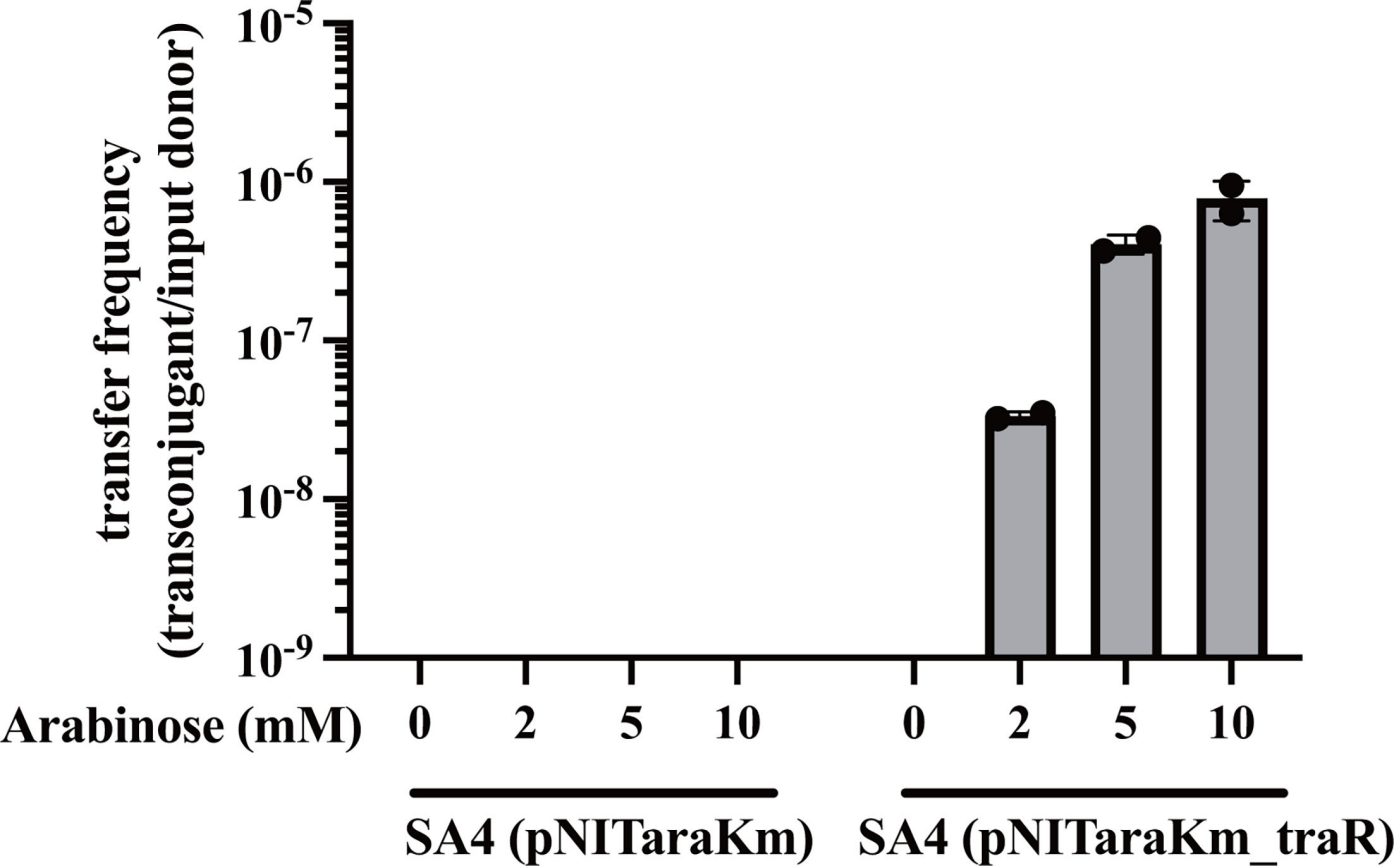
Overexpression of *traR* increased ICE transfer frequency. Donor strains [SA4 (pNITaraKm_traR) and SA (pNITaraKm)] along with a recipient strain (KT2440Gm), were cultured in a liquid medium, then washed, mixed, and spotted onto solid 1/3 LB agar plates with various concentrations of arabinose. Following 24 h of incubation, the cells were collected and spread on selective plates containing Tc and Gm to count transconjugants. The displayed values represent the mean of two independent experiments, with error bars indicating the SD.

To test whether *traR* expression activates the transfer of ICE_KKS102_Tn*4677,* we transformed SA4 with pNITaraKm_traR. SA4 (pNITaraKm_traR) was mated with *P. putida* KT2440Gm (a KT2240 derivative carrying a gentamycin resistance gene for selection) in the presence of different concentrations of arabinose. As shown in Fig. 3, a significant increase in the transfer frequency of ICE_KKS102_Tn*4677* was observed in an arabinose-concentration-dependent manner, showing that the *traR* expression activates the ICE transfer. Colony PCR confirmed that the colonies grown on a plate containing Tc and Gm carried ICE_KKS102_Tn*4677* by colony PCR (data not shown). No transfer was observed in the vector control experiment using SA4 (pNITaraKm).

### Transfer of ICE_KKS102_Tn*4677* is activated in the *bphS* mutant

Conjugal transfer of ICE_KKS102_Tn*4677* in wild-type strain KKS102 has been reported to occur at a very low rate, around 10^−10^, even in SA10 (13). As we have established the activating function of *traR* and *traR* upregulation in SA10, we next reevaluated the transfer frequency of ICE using SA4 and SA10. In our experiment using KT2440Gm as a recipient, we observed no transconjugant when SA4 was used as a donor (transfer frequency <4.2 × 10^−9^). In contrast, when SA10 was used as a donor, transconjugants were obtained at a frequency of 3.8 ± 3.6 × 10^−8^ per input donor cell (Table 2).

**TABLE 2:** Transfer frequency of ICE_KKS102_Tn*4677* in SA4 and SA10

Donor	Recipient	Selection	Frequency/input donor cell
SA4	KT2440Gm	Tc^r^, Gm^r^	<4.2 ± 1.4 × 10^−9^ (*n* = 5)
SA10	KT2440Gm	Tc^r^, Gm^r^	3.8 ± 3.6 × 10^−8^ (*n* = 5)

### TraR overexpression activated retransfer of ICE_KKS102_Tn*4677* in KT2440

We next examined whether the transfer of ICE_KKS102_Tn*4677* in a KT2440-derived transconjugant, KT2440ICE, is activated by *traR* overexpression. KT2440ICE is a KT2440-derived transconjugant that is sensitive to gentamycin and was selected for Cm resistance [an intrinsic phenotype of *P. putida* KT2440 (32)] and Tc resistance. As expected, the mating of KT2440ICE (pNITaraKm_traR) with KT2440Gm resulted in the transfer of ICE. No transfer was observed in the vector control experiment using KT2440ICE (pNITaraKm). Transconjugants were obtained only when the experiment was conducted in the presence of arabinose (Table 3). These results indicated that the TraR target is located on ICE_KKS102_Tn*4677*.

**TABLE 3:** Transfer frequency of ICE_KKS102_Tn*4677* in KT2440ICE

Donor	Recipient	Selection	Frequency/input donor cell
KT2440ICE(pNITaraKm)^*a*^	KT2440Gm	Tc^r^, Gm^r^	<1.0 × 10^−10^ (*n* = 3)
KT2440ICE(pNITaraKm_traR)^*a*^	KT2440Gm	Tc^r^, Gm^r^	8.0 ± 3.1 × 10^−8^ (*n* = 3)

^*a*^ Mating experiment was conducted on a solid medium containing arabinose.

### Excision of ICE by *traR* overexpression

**Fig 4:**
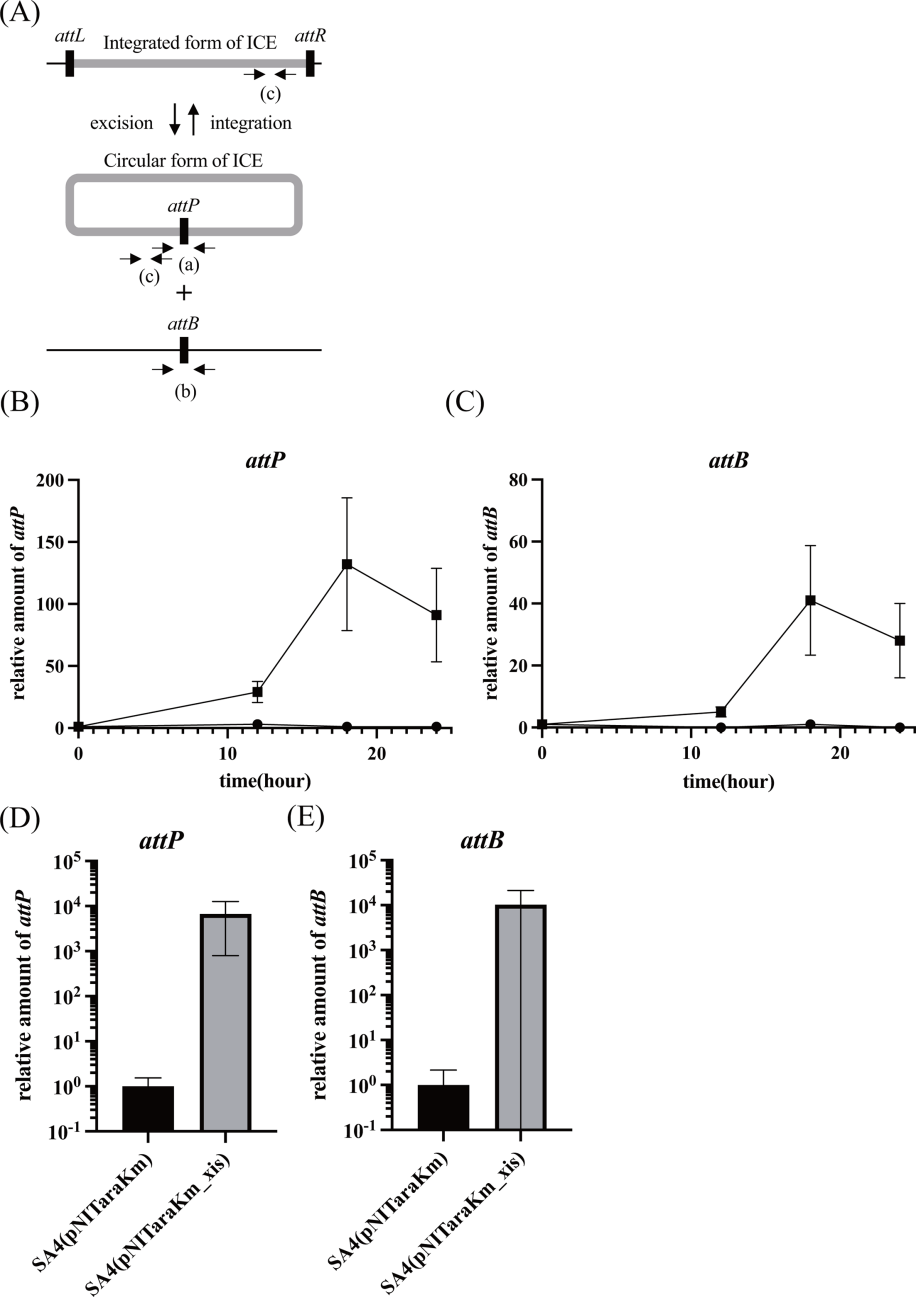
Overexpression of *traR* increased the excised form of ICE. (**A**) Schematic representation of the circular and integrated forms of ICE. Three primer sets were designed to detect the circular form (*attP*, primer set a), a site after excision of ICE (*attB*, primer set b), and integrated and excised forms (primer set c). (**B and C**) ICE excision by *traR* induction. Strains SA4 (pNITaraKm; solid circle) and SA4 (pNITaraKm_traR; solid square) were cultured in liquid 1/3 LB medium, harvested, and spotted onto solid 1/3 LB agar plates containing 10 mM arabinose. At each time point, cells were collected and subjected to qPCR analysis to quantify the amounts of *attP* (**B**) and *attB* (**C**) DNAs. The quantity of DNA was normalized using primers that target both forms of ICE. The values are relative to the value with the initial time point value, which was set as 1. (**D and E**) ICE excision by *xis* induction. Strains SA4 (pNITaraKm) and SA4 (pNITaraKm_xis) were cultured in a liquid medium, harvested, and spotted onto solid 1/3 LB agar plates containing 10 mM arabinose. After 1 h, cells were collected to quantify the amounts of *attP* (**D**) and *attB* (**E**) DNAs.

The increased transfer suggested enhanced ICE excision. Accordingly, we investigated the effect of *traR* overexpression on the copy numbers of *attP* and *attB*, which are formed by ICE excision (Fig. 4). Of note, in our preparative experiment using SA4 (pNITaraKm_traR) grown in liquid media, the increase in the level of excision was very modest and remained less than twofold. However, as we continued our experiments, we found that we could observe a marked increase when we spotted and grew the cells on a solid medium. The conditions were very similar to those we employed in the mating experiments. Even on solid media, both the *attP* and *attB* copy numbers remained relatively low for the initial 12 h of incubation after induction of *traR* expression by spotting the cells on an arabinose-containing medium. Thereafter, the *attP* and *attB* copy numbers relative to those at time 0 increased much more considerably, reaching 130- and 40-fold increases for *attP* (panel B) and *attB* (panel C), respectively.

In one RNA-seq analysis, *traR* induction resulted in a 122-fold increase of NGS reads mapped to the *traR* gene at 1 h after the addition of arabinose. However, no upregulation of *xis* was observed, suggesting that the *traR* function is regulated post-transcriptionally (see the Discussion).

### *traR* overexpression resulted in upregulation of *xis*

**Fig 5:**
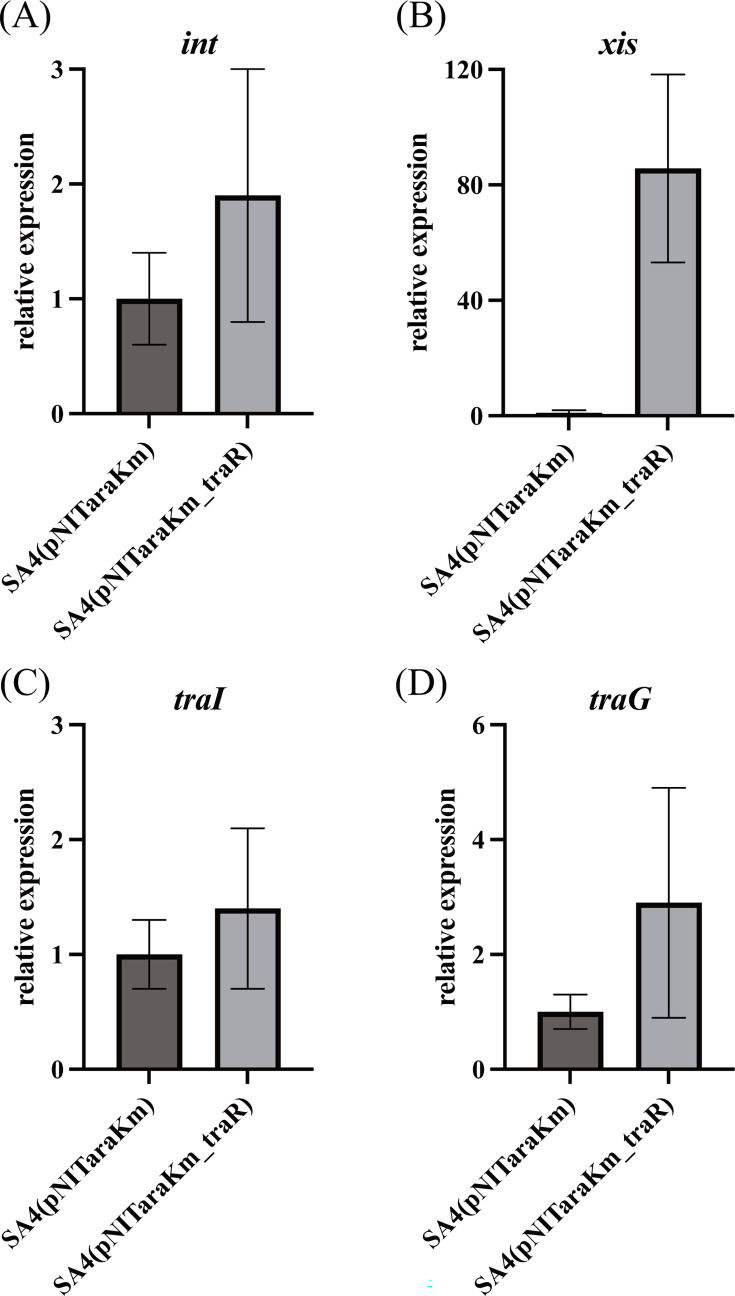
Transcriptional induction of ICE genes by overexpression of *traR*. Two strains, SA4 (pNITaraKm) and SA4 (pNITaraKm_traR), were cultured in a liquid medium, harvested, and spotted onto solid 1/3 LB agar plates containing arabinose. After 18 h, the cells were collected for RNA isolation, and qRT-PCR experiments were conducted for *int* (**A**), *xis* (**B**), *traI* (**C**), and *traG* (**D**). The values are expressed as relative to those of the strain SA4(pNITaraKm), with the *rrn* gene used as the internal standard. The values represent the average of three independent experiments, and error bars indicate SDs.

We investigated whether the transcription levels of several genes of ICE, including the *xis* gene, are upregulated by the overexpression of *traR*. In our initial attempts, we detected no obvious increase in the *xis* transcript after either 1 or 3 h of *traR* induction. On the other hand, we observed a clear increase in the *xis* transcript level after prolonged incubation in the presence of arabinose (see the Discussion for details), which is consistent with the excision frequency data shown above. Figure 5 shows the results of qRT-PCR, conducted using cells after 18 h of incubation on a solid medium. Among the genes tested (*xis*, *int*, *traG*, and *traI*), the transcription level of *xis* showed the greatest increase, i.e., 80-fold, by *traR* induction. The transcription levels of the *traG* and *int* genes increased 2.9-fold (*P*-value = 0.061) and 1.9-fold (*P*-value = 0.054), respectively, while the *traI* level remained unchanged. The transcriptional induction of *traG* and *int* was also not evident 1 h after induction (data not shown).

### Stimulation of excision by *xis* overexpression is not sufficient to activate transfer

As shown in Fig. 4 (panels D and E), overexpression of *xis* resulted in the enhanced excision. To test whether TraR-mediated ICE excision, by upregulation of *xis*, is sufficient for the activation of ICE transfer, the mating assay was conducted using strain SA4 carrying the *xis* expression plasmid pNITaraKm_xis (Table 4). We observed no ICE transfer, indicating that TraR plays a more essential role than the upregulation of *xis* to increase the circular form ICE.

**TABLE 4:** Transfer frequency of ICE_KKS102_Tn*4677*

Donor	Recipient	Selection	Frequency/input donor cell
SA4 (pNITaraKm_xis)^*a*^	KT2440Gm	Tc^r^, Gm^r^	<3.6 ± 0.92 × 10^−9^ (*n* = 3)
SA4 (pNITaraKm)^*a*^	KT2440Gm	Tc^r^, Gm^r^	<5.0 × 10^−9^ (*n* = 3)
SA4 (pNITaraKm_TraR)^*a*^	KT2440Gm	Tc^r^, Gm^r^	3.5 ± 4.5 × 10^−7^ (*n* = 3)

^*a*^ Mating experiment was conducted on a solid medium containing arabinose.

### *In silico* search for TraR-binding sites

**Fig 6:**
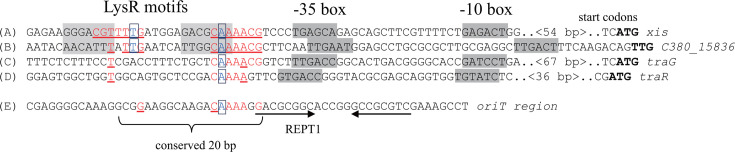
DNA sequences harboring putative TraR-binding sites. A TN_11_A LysR-binding motif, featuring T and A in an interrupted inverted repeat, was found immediately upstream of the predicted *xis* promoter (A). A similar sequence, with 3 mismatches across 14 nucleotides (matching nucleotides are underlined), was identified immediately upstream of the predicted promoter for C380-15836 (B). The half-site was located immediately upstream of the predicted promoters for *traG* (C) and *traR* (D)). This half-site also appears in the 20 bp sequence conserved in the *traF-traI* intergenic region across related ICE elements (E). The start codon for each gene is highlighted in bold. Promoter elements (the −10 and −35 boxes), as predicted by the method described by Mulligan (33), are displayed on a shaded background. T and A in the LysR motif (TN_11_A) are enclosed in boxes. The REPT1 sequence is also indicated.

We explored the DNA sequence upstream of *xis* to identify the binding sites of TraR. As shown in Fig. 6, at the immediate upstream region of the −35 box element of a predicted promoter, we found an interrupted inverted repeat (CGTTT**T**GATGGAGACGC**A**AAACG, designated as BS^xis^) containing the LysR-binding motif of TN_11_A (31). The distance of BS^xis^ from the −35 box of the promoter suggested that this is a canonical promoter regulated by a LysR-type transcriptional regulator. By searching for sequences similar to BS^xis^, we identified a similar putative-binding site (TTTAT**T**GAATCATTGGC**A**AAACG, designated as BS^15836^) upstream of a gene encoding a hypothetical protein (C380_15836). BS^15836^ was also found immediately upstream of a predicted promoter.

A further extensive search for similar sequences revealed a half-binding site upstream of the *traG* putative promoter (CAAAACG, designated as BS^traG^). Additionally, we found CAAAAGG (designated as BS^oriT^) within the well-conserved 20 bp sequence of *oriT* in ICE_KKS102_Tn*4677*, situated upstream of *traI* (see below). Also, a putative *traR* promoter was preceded by a less-conserved half site of CAAAA (designated as BS^traR^). Since half sites of symmetric-binding sites are known to be recognized by a monomer in a dimeric conformation (34), these sites might serve as binding sites for a heterodimer consisting of TraR and an as-yet-unknown factor.

### DNA binding of TraR

To investigate the binding of TraR to the DNA regions upstream of *xis*, we purified N-terminal His-tagged TraR protein and tested its binding to BSxis. Despite considerable efforts, only a very weak interaction was observed between TraR and the DNA fragment containing BSxis. The reason for this weak interaction remains unclear, but it might be attributable to either the stability of the TraR protein or the absence of an unknown inducer. The addition of an N-terminal His-tag is unlikely to be the cause, as the N-terminal His-tagged TraR demonstrated functionality in increasing the ICE circular form. TraR binding to other putative binding sites was also not successful.

### pICEoriT was transferred to KT2440 by *traR* overexpression

To better understand the ICE transfer, it is essential to identify *oriT* of ICE_KKS102_Tn*4677*. Because the intergenic regions upstream of *traI* and downstream of *traF* from related ICEs exhibited a higher level of sequence conservation than other intergenic regions, we predict that the *oriT* of ICE_KKS102_Tn*4677* is located in this intergenic region. To test this, a plasmid pICEoriT was constructed by cloning the intergenic region (463 bp) into a broad host range vector, pBBR1-MCS5 (29). The pICEoriT plasmid was transferred to KT2440 not from SA7 or SA17 but from SA4 and SA10, with a frequency two orders of magnitudes higher for SA10 (Table 5), showing that *traR* activates the transfer of pICEoriT and is essential for transfer. Furthermore, the overexpression of *traR* expressed from pNITaraKm_traR resulted in a higher frequency value of 1.9 × 10^−3^. The *traR* activation of transfer of pICEoriT showed that *xis*-induction leading to ICE excision is not the sole role of TraR in transfer activation.

**TABLE 5:** Transfer frequency of pICEoriT

Donor (plasmid) [relevant host genotype]	Recipient	Selection	Frequency/input donor cell
SA4 (pICEoriT)	KT2440	Cm^r^, Gm^r^	2.7 ± 4.2 × 10^−7^ (*n* = 2)
SA7 (pICEoriT) [∆bphR]	KT2440	Cm^r^, Gm^r^	<2.9 × 10^−8^ (*n* = 2)
SA10 (pICEoriT) [∆bphS]	KT2440	Cm^r^, Gm^r^	1.1 ± 0.7 × 10^−5^ (*n* = 2)
SA17 (pICEoriT) [∆bphS∆traR]	KT2440	Cm^r^, Gm^r^	<7.8 × 10^−8^ (*n* = 2)
SA4 (pBBR1-MCS5)	KT2440	Cm^r^, Gm^r^	<1.1 × 10^−9^ (*n* = 2)
SA4 (pICEoriT) (pNITaraKm)	KT2440	Cm^r^, Gm^r^	<5.8 × 10^−9^ (*n* = 2)
SA4 (pICEoriT) (pNITaraKm_traR)	KT2440	Cm^r^, Gm^r^	1.9 × 10^−3^ (*n* = 2)

### Identification of the essential *oriT* sequence

**Fig 7:**
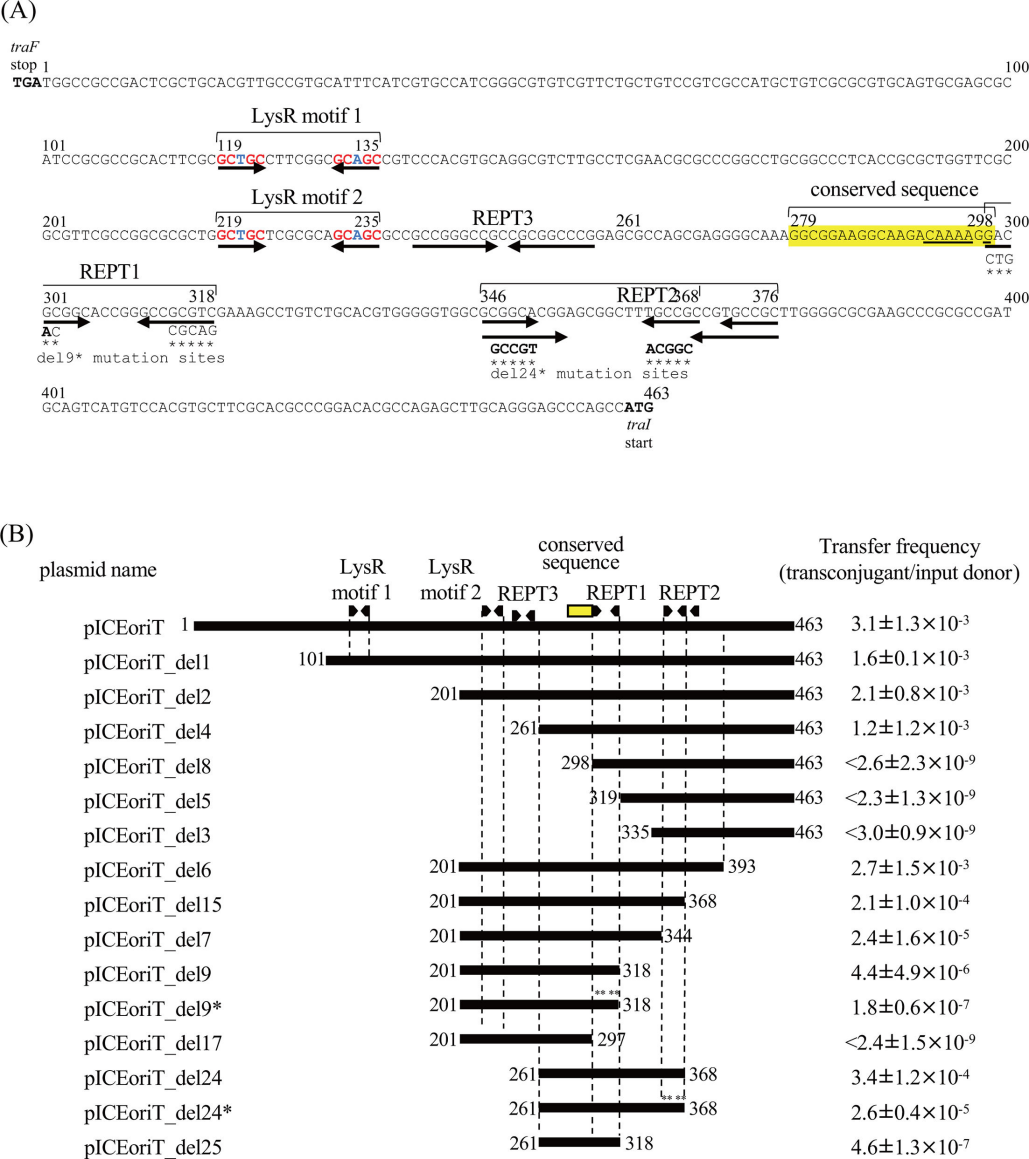
Deletion analysis of the *traF-traI* intergenic region for *oriT* function. (**A**) DNA sequence of the *traF-traI* intergenic region. The conserved 20 bp sequence (highlighted in yellow), putative LysR motif, and repeat elements (arrows; REPT1, 2, and 3) are indicated. Asterisks mark the mutated bases in pICEoriT_der9* and pICEoriT_der24*, and the sequences after mutation are shown above the asterisks. The mutations in pICEoriT_der9* and pICEoriT_der24* preserved the inverted repeat, although there was one nucleotide mismatch in pICEoriT_der9* (drawn in bold). The possible TraR association site in the conserved 20 bp sequence is underlined. (**B**) Deletion analysis of the *oriT* region. The horizontal bars indicate DNA regions cloned into pBBR1-MCS. *oriT* plasmid names and transfer frequencies are shown to the left and right of the bars, respectively.

In the *oriT* region, we found two LysR binding motifs (LysR motifs 1 and 2), a conserved 20 bp sequence (see Fig. S2), and three repetitive elements (REPT1, REPT2, and REPT3; Fig. 7, panel A). To identify the minimum *oriT* region, we constructed a series of deletion plasmids of pICEoriT and measured the transfer frequency under *traR*-overexpressing conditions. As shown in panel B in Fig. 7, the transfer frequency of pICEoriT (3.1 × 10^−3^) was roughly unchanged when the *oriT* region was deleted from the left side (*traF* side) up to position 261 (see del4 in Fig. 7), showing that the two LysR motifs are not necessary for *oriT* function. The frequency was decreased to an undetectable level when the *oriT* region was deleted to position 298 (del8). When deleted from the right side (*traI* side), deletion to position 393 (del6) did not affect the frequency, but when the REPT2 was deleted, the frequency decreased by two orders of magnitude (del7). The deletion to just before the REPT1 did not abolish transfer (del9), but the deletion of REPT1 resulted in an undetectable level of frequency (del17). The del25 (carrying the region from position 261–318 and lacking REPT3) with the conserved 20 bp sequence and REPT1 maintained *oriT* activity. The sequences of REPT1 and REPT2 themselves are not essential because the substitution of bases (del9* and del24*) had some effects but did not abolish the *oriT* function. Since the mutations in either del9* or del24* contained the inverted repeat (see Fig. 7A), it seems that the presence of the inverted repeats, rather than the specific nucleotide sequence, is important. Taking these results together, we concluded that the essential *oriT* functional sequence lies between positions 261 and 318 (58 bp), where a conserved 20 bp sequence and REPT1 are located, and the regions around RPET2 have some effect in enhancing transfer frequency.

## DISCUSSION

**Fig 8:**
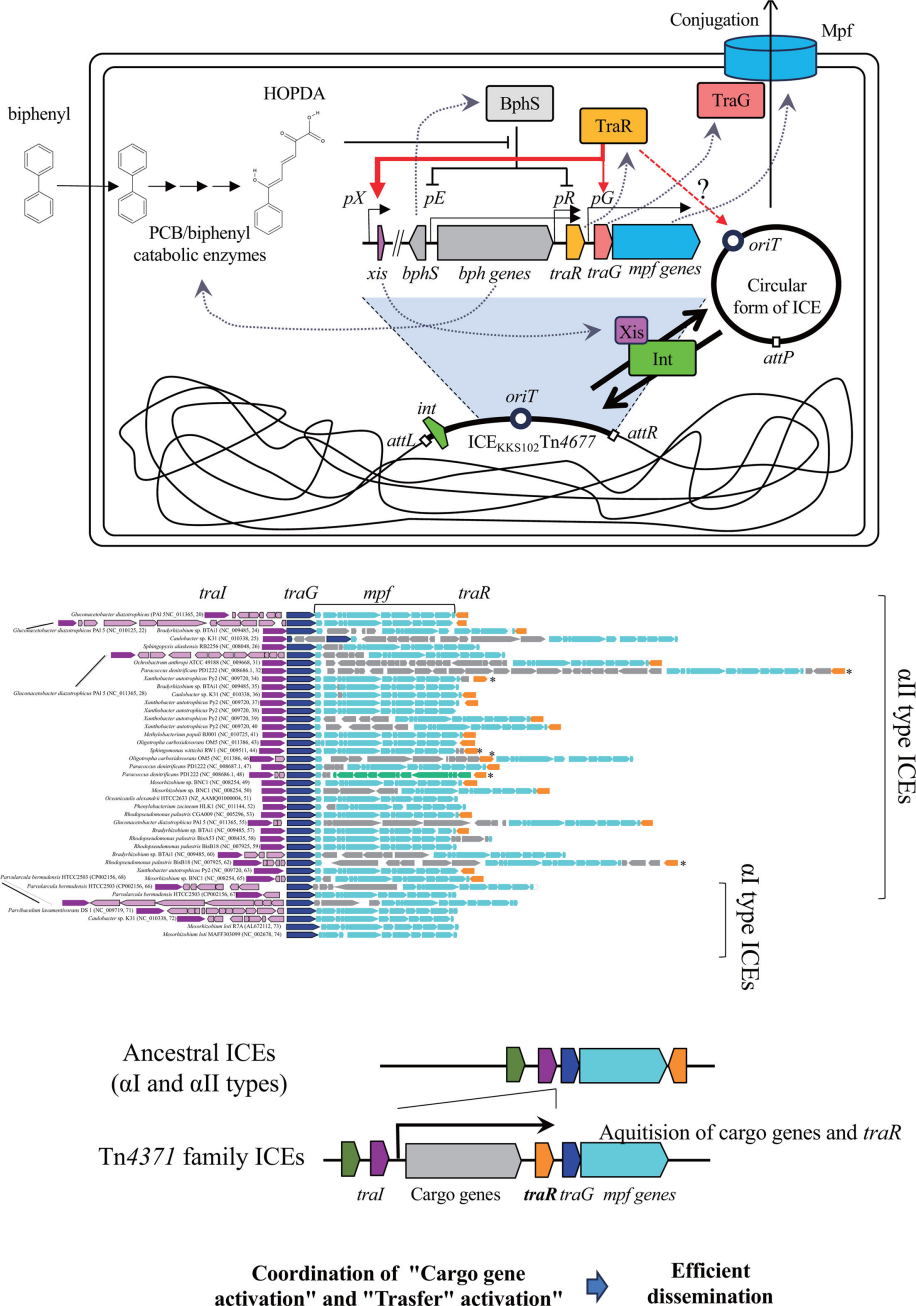
A model illustrating the activation of ICE transfer in response to biphenyl. In the presence of PCBs/biphenyl, the intermediate compound HOPDA mitigates repression by BphS, leading to the upregulation of the *pE* and *pR* promoters, which in turn results in the expression of TraR. TraR enhances the expression of *xis*, facilitating ICE excision. Additionally, TraR upregulates the *pG* promoter, inducing the expression of TraG (the coupling protein) and the formation of the mating pair. Consequently, ICE transfer is activated in the presence of PCBs/biphenyl, with TraR playing the central role. The *traR* gene direction and location situated downstream of cargo genes are conserved characteristics among Tn*4371* family ICEs. This gene organization allows the ICE transfer under environmental conditions that activate cargo gene transcription. This would facilitate the efficient dissemination of ICE, as the cargo genes are likely to instantly confer fitness advantages to newly ICE-acquiring bacteria in that environment. Notably, ancestral ICEs, namely types αI and αII, do not possess the cargo and *traR* genes at the corresponding locations. The gene organization map shows the *traI-traG*-mpf regions of a total of 41 ICEs, including 7 αI type and 36 αII type ICEs from the report by Ohtsubo et al. (13). Each species name is followed by, in parentheses, the accession number and the sequence number assigned in that report (13). Gene symbols are colored as follows: *traI* in purple, *traG* in dark blue, *mpf* genes in cyan, *traR* in orange, cargo genes between *traI* and *traG* in pink, and other cargo genes in gray. The *traR* gene is predominantly located downstream of the *mpf* genes in reverse orientation. *traR* genes with different localizations are marked with asterisks.

In this study, we established that *traR*, co-transcribed with the *bph* operon, is the activator of ICE_KKS102_Tn*4677* transfer and identified the essential *oriT* sequence. We also elucidated aspects of the mechanisms underlying *traR*-mediated ICE transfer activation, in which TraR activates *xis* transcription, leading to ICE excision. Also, upregulation of the *traG* promoter by TraR was demonstrated. Our study sheds light on the molecular and genetic basis of the dissemination of the Tn*4371* family of ICEs (Fig. 8).

### *traR* transcription is initiated from *pE* and *pR* promoters

*traR* is transcribed from two promoters, *pE* and *pR*. The *pE* promoter is regulated by BphS (16), and in this study, we demonstrated an increase in the *traR* transcript in the absence of *bphS*. In addition, replacing the *pE* promoter with a constitutive strong promoter [the *tac* promoter (30)] resulted in an increase in the *traR* transcript. These findings clearly showed that *traR* is transcribed from *pE* under the control of BphS, which allows the *bph* operon for PCB/biphenyl degradation to be upregulated only in the presence of the degradation substrate. We also found that the *pR* promoter is upregulated in the absence of *bphS* (Fig. 2B).

However, no putative BphS-binding sites have been found in the upstream region of *traR*, and the overexpression of TraR did not lead to an increase in *pR* promoter activity. At present, the reason why *pR* activity increases in the *bphS* mutant remains unclear.

### Implications of ICE-transfer activation by the *bph* operon substrate

The regulatory system, which links the availability of the *bph* degradation system’s substrate to the active horizontal transfer of ICE, suggests a strategic advantage in the dissemination of ICE_KKS102_Tn*4677*. This system enhances the transfer of cargo *bph* genes to surrounding bacteria in environments where these genes confer a fitness advantage, potentially playing a crucial role in ICE dissemination. Such a mechanism would ensure the effective spread of the ICE, as bacterial cells in these environments are more likely to benefit from the acquired capabilities.

A wealth of studies have highlighted the diverse mechanisms controlling bacterial horizontal gene transfer. As for the self-transmissible chlorocatechol degradative *clc* genes of *Pseudomonas knackmussii* strain B13, the *clc* element’s integrase gene expression was significantly stimulated by growing cultures on 3-chlorobenzoate (35). In addition, InrR from the *clc* element exerts bistable and stochastic regulation of the excision and transfer of the *clc* element, whose expression involves stationary sigma factor RpoS (36, 37). In *Bacillus subtilis*, the conjugation of a plasmid, pLS20, is inhibited by high levels of the quorum signaling peptide, which can be beneficial in preventing unnecessary gene transfer in densely populated environments (38). In a study by Beaber et al., bacterial SOS response induced by DNA damage was shown to increase the frequency of transfer of the SXT element, an ICE derived from *Vibrio cholerae* (39).

Our finding is that *traR*, whose transcription is co-upregulated with the conditional activation of cargo *bph* genes, activates the horizontal transfer of ICE_KKS102_Tn*4677* and is another good example of how the horizontal transfer of mobile genetic elements is regulated.

### Delay of *xis* upregulation after *traR* induction

We observed a considerable delay in the increase of *xis* transcription and the excision of ICE following the induction of *traR* with arabinose. In bacterial transcription induction, responses typically occur within a very short time span. For instance, with the pBAD promoter, transcription is known to increase within a short period after arabinose addition (40). However, when we induced *traR* expression with arabinose, we did not observe an increase in *xis* transcription within the usual time scale, while NGS data indicated a 122-fold increase in the induction of the *traR* transcript after the addition of arabinose.

Several factors might contribute to this delayed induction, although the precise reasons remain unclear. One possibility is that the stability of TraR or translation efficiency of *traR* is regulated during different growth phases as suggested from the three distinct growing phases of KKS102 (19). Alternatively, it is worth noting that TraR is a LysR-type transcription factor, which might require specific inducing chemicals for transcription activation. The unknown inducer might be generated at the very late stage of growth. Currently, the cues for identifying the unknown inducer are quite limited. However, the fact that *traR*-mediated ICE excision did not occur in liquid culture may provide some hints. The unknown signal may be related to the stable contact with other cells through the formation of T4SS, which might readily break from the cell surface (14). Further investigation is needed to fully understand the delay in TraR-mediated *xis* activation.

### Mechanisms of ICE transfer activation by TraR

In this study, we showed that TraR activated *xis* transcription and resulted in enhanced ICE excision; however, excision alone was not sufficient for the ICE transfer to occur. The plasmid cloned with *oriT* (pICEoriT) required *traR* overexpression for transfer. In addition, while *xis* overexpression did increase the circular form of ICE, it did not result in ICE transfer. The transfer activation of ICE_KKS102_Tn*467*7 in KT2440 by *traR* overexpression indicates that other TraR targets are located on the ICE. Although TraR overexpression induced a modest threefold increase in *traG* expression (Fig. 5), this upregulation suggests that TraR overexpression plays a crucial role in the activation of transfer. The presence of a putative TraR half-binding site very close to the *traG* promoter (*pG* promoter) suggests that the *pG* is under the control of TraR. Our preparative RNA-seq analysis (*n* = 1) showed that very shortly after *traR* induction (1 h), transcripts of the *traG* and *mpf* genes increased 1.9-fold. However, in the subsequent attempt, these genes were not upregulated after 1 h possibly due to slight unintended differences in the experimental conditions, suggesting that an unknown-inducing signal, which was present in the initial attempt, is required to upregulate *pG*. Identifying the yet-unidentified TraR effector would lead to a better understanding of TraR-mediated *pG* activation, as well as of the overall mechanism of TraR-mediated ICE transfer activation.

### Possible role of TraR as an accessory protein

Another possible target of TraR is *oriT*. Although an *oriT* is a cis-acting DNA region required for the conjugation of plasmids and ICEs, it had not been identified in the ICEs of the Tn*4371* family. In this study, we experimentally demonstrated that the intergenic region between the *traI* and *traF* genes on ICE_KKS102_Tn*4677* functions as an *oriT*. This region was not detected by oriTfinder (41), a software tool designed for predicting *oriTs* from DNA sequence information, indicating that the *oriT* is distinct from previously identified *oriTs* and represents a novel *oriT*.

The *nic* site is most likely located within the 20 bp yellow-highlighted conserved region because this area is required for DNA transfer (Fig. 7), and *nic* sites typically occur around 10 nucleotides away from an inverted repeat (42, 43). The mutations in REPT1 resulted in a 24-fold decrease in the transfer frequency (see pICEoriT_del9* in Fig. 7B). The fact that the transfer was not completely abolished might be due to the mutations we introduced; the mutated bases could still form an inverted repeat, although there was one nucleotide mismatch in the eight nucleotides. The core of *oriT* thus consists of a 20 bp conserved sequence and an inverted repeat, where the presence of the inverted repeat, rather than the specific nucleotide sequence, is important.

Within this 20 bp sequence, we found a sequence of CAAAAGG, a half site of the possible TraR-binding sequence found upstream of *xis*. For some LysR-type transcriptional regulators, including CbnR, CysB, AlsR, and OccR, the bending of DNAs upon binding to their binding site has been reported (44–47). Further *in vivo* and *in vitro* studies are required to confirm whether TraR binds to this sequence and potentially bends DNA around *oriT*, acting as an accessory protein to aid TraI’s nicking function at this site.

Miyazaki et al. (48) reported on the presence of dual *oriT* sites in ICEclc, suggesting that our ICE might also have multiple *oriT* sites. To confirm this, it would be necessary to delete the identified *oriT* of our ICE and observe whether transfer still occurs (48).

### *int* gene upregulation might be the result of circular ICE formation

The modest twofold increase in the *int* gene transcription level might have resulted from overexpression of *xis* and subsequent formation of circular ICE. As described previously, the *int* gene is highly transcribed in the circular form (13). A strong promoter located close to the right boundary of ICE transcribes the *int* gene upon forming the circular form. Therefore, if a minor fraction of cells undergoes ICE excision due to *traR* overexpression, it would be possible for the overall *int* transcript levels to increase to this extent.

### Role of TraR in the emergence of Tn*4371* family ICEs

A phylogenic analysis of Tn*4371* family ICEs and related ICEs outside of the Tn*4371* family (types αI and αII) using TraG amino acid sequences showed that the branch lengths among Tn*4371* family ICEs are shorter than those among the related elements from proteobacteria or plasmids (13), suggesting rapid dissemination of Tn*4371* family ICEs. In addition, it seemed that Tn*4371* family ICEs have emerged from ancestral ICEs with gene organization of types αI and αII (13).

In most of the Tn*4371* family ICEs, which are predominantly found in β and γ proteobacteria, the direction of genes in the accessory region 3 is toward the *traR and mpf* genes (Fig. 8), i.e., cargo gene transcription is most likely coordinated with *traR* transcription. Figure S3 shows that 30 out of 32 ICEs found in the Tn*4371* family (13) exhibit this organization. On the other hand, *traR* is absent from this location in Tn*4371*-related ICEs of types αI and αII (Fig. 8), which were collected based on the similarity of encoded TraG to TraG from KKS102 (13). From an evolutionary perspective, with this organization of Tn*4371* family ICEs, the incorporation of a new cargo gene into accessory region 3 would readily set up this coordinated transcription. This coordination would be advantageous for the ICE dissemination because the environmental conditions under which cargo genes are activated are likely the conditions where the ICE gives the fitness advantage to the host bacteria, thereby providing more opportunities to increase the copy number of ICEs. Although how ICEs acquire new cargo genes remains totally unknown, we propose that the recruitment of the *traR* gene in its current position to the ancestral ICE has established the ICE Tn*4371* family by enabling efficient dissemination due to the coordinated transcription of cargo genes and the *traR* gene.

## Data Availability

All sequence data have been deposited in the Short Read Archive under the following accession numbers: SAMN42563764, SAMN42563765, SAMN42563766, and SAMN42563767.

## SUPPLEMENTAL MATERIAL

Fig. S1 spectrum.00607-24-s0001.pdf Promoter-lacZ integration system used.

Fig. S2 spectrum.00607-24-s0002.pdf Alignment of oriT regions from Tn4371 family ICEs.

Fig. S3 spectrum.00607-24-s0003.pdf Gene organization of traR and cargo genes of Tn4371 family ICEs.

Supplemental material spectrum.00607-24-s0004.docx Supplemental figures reference list.

Table S1 spectrum.00607-24-s0005.pdf Synthetic DNAs used.

ASM does not own the copyrights to Supplemental Material that may be linked to, or accessed through, an article. The authors have granted ASM a non-exclusive, world-wide license to publish the Supplemental Material files. Please contact the corresponding author directly for reuse.
