# Supplementary material for: Evolution of the Tn4371 ICE family: traR-mediated coordination of cargo gene upregulation and horizontal transfer
Source: Microbiol Spectr. 2024 Sep 12;12(10):e00607-24. doi: 10.1128/spectrum.00607-24 (PMC11448139; doi:10.1128/spectrum.00607-24)
Supplement: Fig. S1 — Promoter-lacZ integration system used. [file spectrum.00607-24-s0001.pdf]

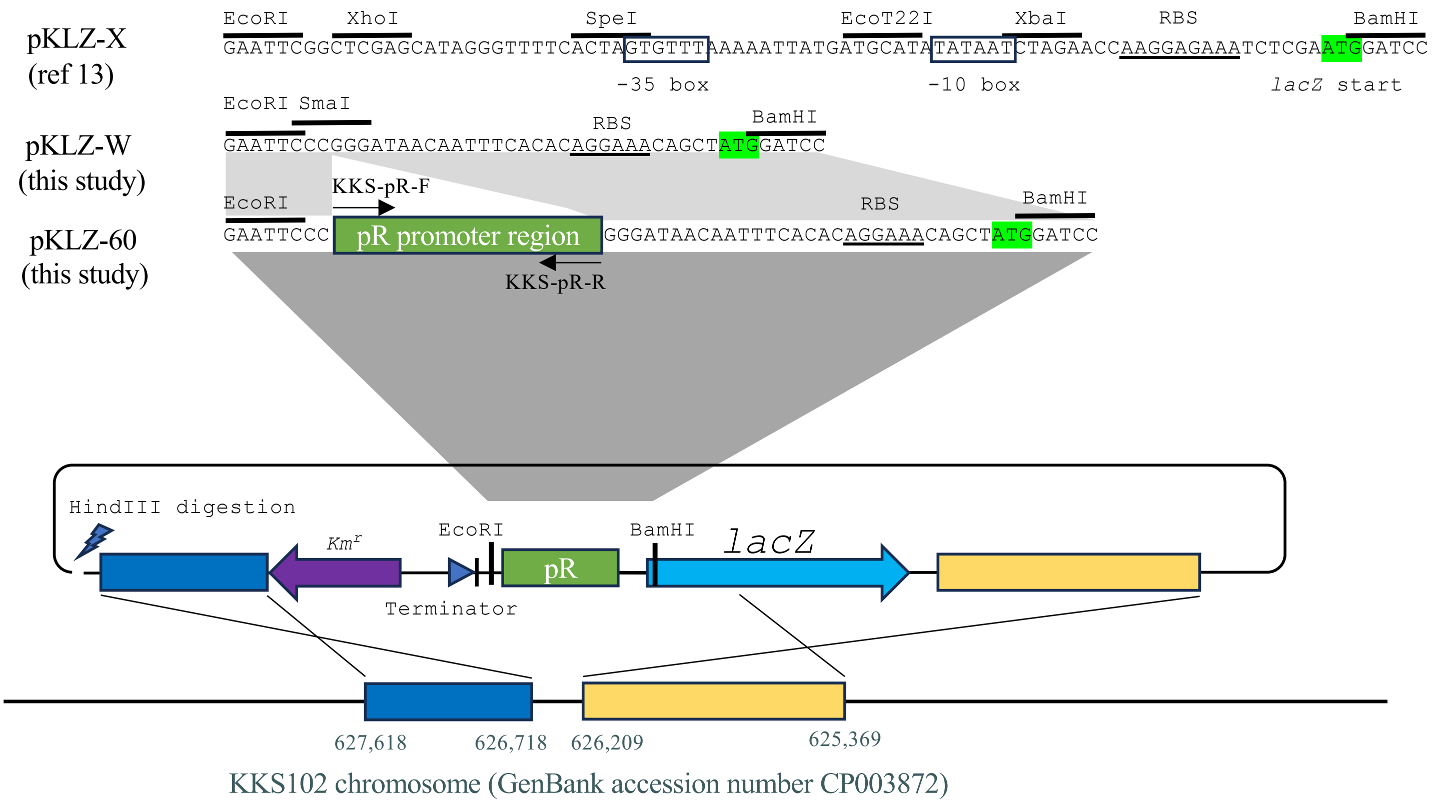

Fig. S1 Promoter-*lacZ* integration system used in this study.

The plasmid pLZZ60 carries the pR promoter in the SmaI site of pKLZ60. The HindIII digest of pKLZ60 was introduced into KKS102 to integrate the pR-lacZ construct into the chromosome of KKS102.
