# Supplementary figures and images for: Evolution of the Tn4371 ICE family: traR-mediated coordination of cargo gene upregulation and horizontal transfer
Source: Microbiol Spectr. 2024 Sep 12;12(10):e00607-24. doi: 10.1128/spectrum.00607-24 (PMC11448139; doi:10.1128/spectrum.00607-24)

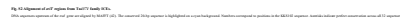

Supplement: Fig. S2 — Alignment of oriT regions from Tn4371 family ICEs. [file spectrum.00607-24-s0002.pdf]
