# Supplementary material for: Evolution of the Tn4371 ICE family: traR-mediated coordination of cargo gene upregulation and horizontal transfer
Source: Microbiol Spectr. 2024 Sep 12;12(10):e00607-24. doi: 10.1128/spectrum.00607-24 (PMC11448139; doi:10.1128/spectrum.00607-24)
Supplement: Fig. S3 — Gene organization of traR and cargo genes of Tn4371 family ICEs. [file spectrum.00607-24-s0003.pdf]

Fig.S3 Satoshi Matsumot et al.

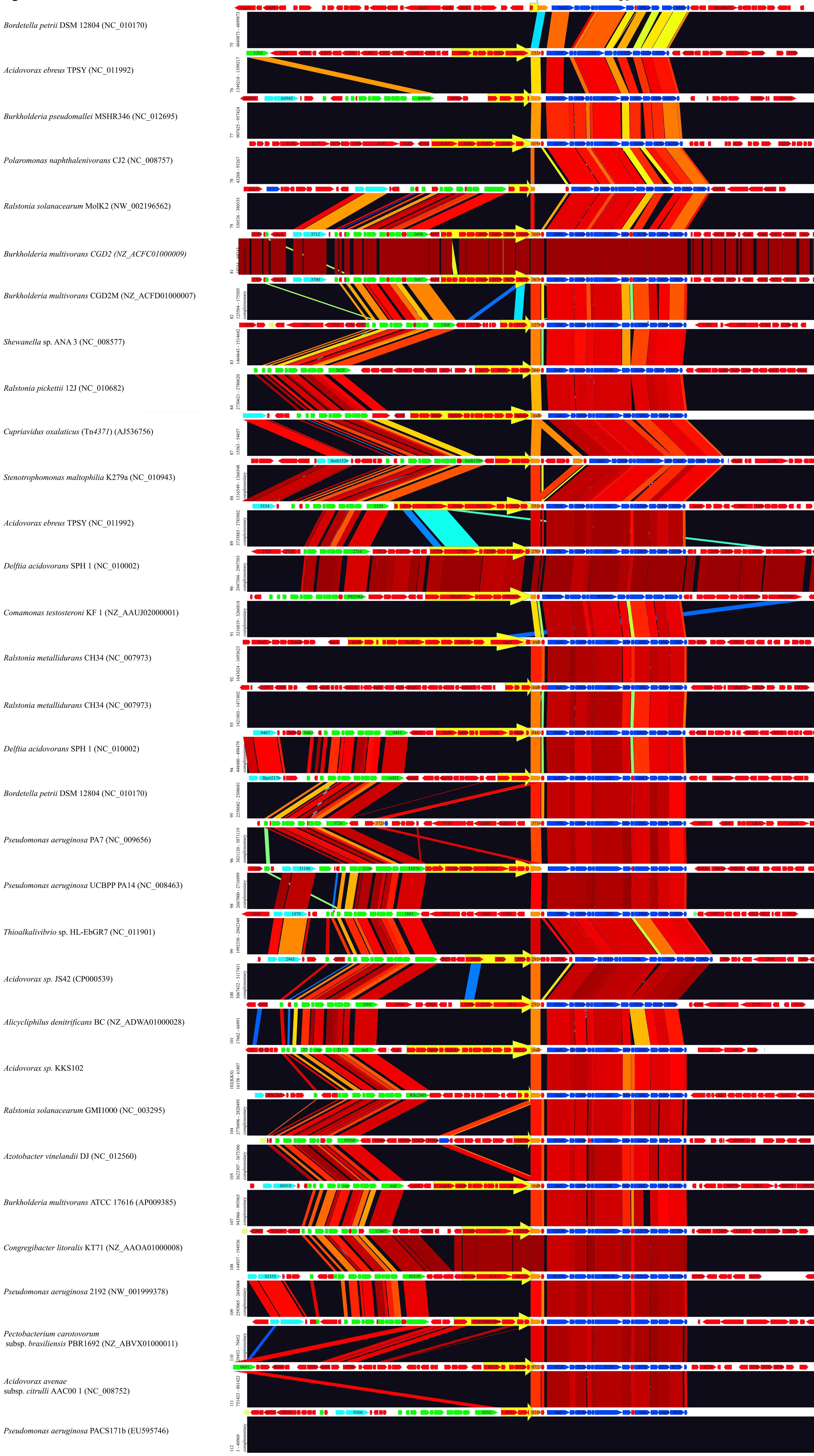

Fig. S3 Gene organization of *traR* and cargo genes of Tn*4371* family ICEs
Alignment of all 32 founding members of the ICE Tn*4371* family. In each sequence, the *traR* gene (orange) is in the center and heading right. Genes within the mpf cluster are represented in blue, those within the *traI* gene block in green, and the *parB* block gene in cyan. The cargo genes whose transcription is likely coupled with *traR* are highlighted by large yellow arrows. The numbers displayed on the left (75 to 112) correspond to identifiers assigned to each ICE as referenced in Ohtsubo et al. (1). The range of DNA sequences utilized for comparison are indicated (1). The alignment was created by using the CompareSequences tool of GenomeMatcher software (2). The comparison was carried out using BLASTP with the parameter "-word_size 3". Hits with more than 40% identity and a query coverage greater than 50% are displayed.
