# Supplementary material for: Evolution of the Tn4371 ICE family: traR-mediated coordination of cargo gene upregulation and horizontal transfer
Source: Microbiol Spectr. 2024 Sep 12;12(10):e00607-24. doi: 10.1128/spectrum.00607-24 (PMC11448139; doi:10.1128/spectrum.00607-24)
Supplement: Supplemental material — Supplemental figures reference list. [file spectrum.00607-24-s0004.docx]

Supplemental Reference List

1. Ohtsubo Y, Ishibashi Y, Naganawa H, Hirokawa S, Atobe S, Nagata Y, Tsuda M. 2012. Conjugal Transfer of Polychlorinated Biphenyl/Biphenyl Degradation Genes in *Acidovorax* sp. Strain KKS102, Which Are Located on an Integrative and Conjugative Element. J Bacteriol 194:4237-48.

2. Ohtsubo Y, Ikeda-Ohtsubo W, Nagata Y, Tsuda M. 2008. GenomeMatcher: a graphical user interface for DNA sequence comparison. BMC Bioinformatics 9:376.
