# Supplementary material for: Evolution of the Tn4371 ICE family: traR-mediated coordination of cargo gene upregulation and horizontal transfer
Source: Microbiol Spectr. 2024 Sep 12;12(10):e00607-24. doi: 10.1128/spectrum.00607-24 (PMC11448139; doi:10.1128/spectrum.00607-24)
Supplement: Table S1 — Synthetic DNAs used. [file spectrum.00607-24-s0005.pdf]

Table S1 Synthetic DNAs used in this study. Synthetic DNAs were purchased either from Eurofins Genomics Co., Ltd., or Hokkaido System Science (indicated by H; Hokkaido, Japan)

oligo nucleotides	sequence 5' → 3'	usage / note
SM141	GAAGGAGATATACATATGCACCACCACCACCACCACATG GAACTGCGACACCTG	construction of pET22b(+)_His-traR
SM142	GTGGTGGTGCTCGAGTCATTGCATCAGCTCCTG	construction of pET22b(+)_His-traR
SM083	TTTGGCTCGAGATCTATGCGTCCCGCTCCTTTG	construction of pNITaraKm_xis
SM084	AAAAGCTTCGAATTCTCAGCGCGCACGGCTG	construction of pNITaraKm_xis
PKLZ-W-a	AATTCCCGGGATAACAATTTCACACAGGAAACAGCTATG	H, construction of pKLZ-W
PKLZ-W-b	GATCCATAGCTGTTTCCTGTGTGAAATTGTTATCCCGGG	H, construction of pKLZ-W
KKS-pR-F	TGTCGAAACTCAGCCGCGCC	H, construction of pKLZ60
KKS-pR-R	GCATCGAGCTCCCGTTTGG	H, construction of pKLZ60
traR-GA-F	CGTTTTTTTG*CTCGAG*AGGGAGCTCGATGCATGG	construction of pNITaratraR
traR-GA-R	CAAAAGCTTC*GAATTC*AGGATGCTTATTTTCATTGC	construction of pNITaratraR
PNIT5548-FAM	CTGACACCCTCATCAGTGCCAA	preparation of FAM-labeled xis promoter region DNA
PNIT5041-FAM	GTGAGAAATCACCATGAGTGACG	preparation of FAM-labeled xis promoter region DNA
M4out-FAM	GCTGCAAGGCGATTAAG	preparation of FAM-labeled oriT region DNA
RVout-FAM	GGCTCGTATGTTGTGTG	preparation of FAM-labeled oriT region DNA
SM484	GAATGGCAAAAGCTTGACCTGGCCTCCGATTTCTC	preparation of FAM-labeled traG promoter region DNA
SM485-FAM	TCCTGCCCTTGCTGGTGG	preparation of FAM-labeled traG promoter region DNA
SM103	ATACACTCCGCTAGCCACAACTGTCGCCGCTTG	construction of pNITxisup
SM104	GAATGGCAAAAGCTTGAGATGAACCTCCATCGGTTG	construction of pNITxisup
SM015	CGTAGGTGATGGCATCGTCAC	qRTPCR for traR
SM016	ATAGCGCAGCAGTTCTTCCAGG	qRTPCR for traR
SM019	ATGCTGACGAGTGGCGAAC	qRTPCR for rrn
SM020	TGAGATCGTATGCGGTATTAGCA	qRTPCR for *rrn*
SM021	TTAAGCCAGAGCATGGCATCCGCAC	Amplification of left region of ICE
SM022	CGGCCCTGAAGTAGATGTTG	Amplification of left region of ICE
SM023	AGAAGCTTGGGCGCTTCGAC	Amplification of right region of ICE
SM024	GTCCTTTCTGCTTTCGCCAC	Amplification of right region of ICE
SM025	CCTCATCCAAGTCCATCGTTAGC	quantification of *attP* site
SM026	CCAAGTCCTTGCCACTATTGACC	quantification of *attP* site
SM027	CCACTCGGGCATTACATGATGC	quantification of both forms of ICE

SM028	GGTCAATAGTGGCAAGGACTTGG	quantification of both forms of ICE
SM349	CTTGACATGATTGAATCGTAGTG	quantification of *attB* site
SM350	AGGTATGCGCTCAGCAG	quantification of *attB* site
SM041	CATTCGAGCTGACCTTGCAC	qRTPCR for *traG*
SM042	GAGCGTTCGTTGTCCGAC	qRTPCR for *traG*
SM105	GCCGCTGTCTCAACTG	qRTPCR for *xis*
SM106	GGCCGAACTTGCGAAAG	qRTPCR for xis
SM074	CTGACGAATGCACCTCG	qRTPCR for *int*
SM075	CCACGCCTGATAGTAGG	qRTPCR for *int*
SM141	GAAGGAGATATACATATGCACCACCACCACCACCACATGGAACTGCGACACCTG	qRTPCR for *traI*
SM142	GTGGTGGTGCTCGAGTCATTGCATCAGCTCCTG	qRTPCR for *traI*
SM152	CGGTATCGATAAGCTTATCCGCGCCGCACTTCG	construction of pICEoriT_del1
SM153	CGGTATCGATAAGCTTGCGTTCGCCGGCGCGCTG	construction of pICEoriT_del2, 6, 7, 9, 9*, 15, 17
SM154	CGGTATCGATAAGCTTGTGGGGGTGGCGCGGCAC	construction of pICEoriT_del3
SM155	CGGGCTGCAGGAATTCCATGGCTGGGCTCCCTGC	construction of pICEoriT_del1, 2, 3, 4, 5, 8
SM180	CGGTATCGATAAGCTTCGCCAGCGAGGGGCAAAG	construction of pICEoriT_del4, 24, 24*, 25
SM181	CGGTATCGATAAGCTTGAAAGCCTGTCTGCACG	construction of pICEoriT_del5
SM182	CGGGCTGCAGGAATTCGGGCTTCGCGCCCCAAGC	construction of pICEoriT_del6
SM183	CGGGCTGCAGGAATTCCACCCCCACGTGCAGACAG	construction of pICEoriT_del7
SM191	CGGTATCGATAAGCTTGACGCGGCACCGGGCCGCGTCGAAAG	construction of pICEoriT_del8
SM204	CGGGCTGCAGGAATTCGACGCGGCCCGGTGCCGCGTC	construction of pICEoriT_del9
SM205	CGGGCTGCAGGAATTC<u>CTGCG</u>GGCCCGGTGCC<u>CTCAG</u>CTTTGTCTTG	construction of pICEoriT_del9*, mutation sites are underlined.
SM231	CGGGCTGCAGGAATTCGACGCGGCCCGGTGCCGCGTCCTTTTG	construction of pICEoriT_del25
SM239	CGGGCTGCAGGAATTCGCGGCAAAGCCGCTCCGTG	construction of pICEoriT_del15, 24
SM241	CGGGCTGCAGGAATTCTCTTTTGTCTTGCCTTCCGCCTTTG	construction of pICEoriT_del17
SM261	CGGGCTGCAGGAATTCG<u>GCCGT</u>AAGCCGCTCCG<u>ACGGC</u>CGCCACCCCCACGTGCAG	construction of pICEoriT_del24*, mutation sites are underlined.
